# The Extended Synaptotagmins of *Physcomitrium patens*

**DOI:** 10.3390/plants14071027

**Published:** 2025-03-25

**Authors:** Alexander Kaier, Maria Ntefidou

**Affiliations:** 1 Division of Biochemistry, Department of Biology, Friedrich-Alexander-University Erlangen-Nuremberg, 91058 Erlangen, Germany; alexander.kaier@fau.de; 2 Division of Cell Biology, Department of Biology, Friedrich-Alexander-University Erlangen-Nuremberg, 91058 Erlangen, Germany

**Keywords:** extended synaptotagmins, membrane contact sites, lipid transport proteins, tip growth, plasmodesmata, *Physcomitrium patens*, bryophytes

## Abstract

Membrane contact sites (MCSs) between the endoplasmic reticulum and the plasma membrane enable the transport of lipids without membrane fusion. Extended Synaptotagmins (ESYTs) act at MCSs, functioning as tethers between two membrane compartments. In plants, ESYTs have been mainly investigated in *A. thaliana* and shown to maintain the integrity of the plasma membrane, especially during stress responses like cold acclimatization, mechanical trauma, and salt stress. ESYTs are present at the MCSs of plasmodesmata, where they regulate defense responses by modulating cell-to-cell transfer of pathogens. Here, the analysis of ESYTs was expanded to the bryophyte *Physcomitrium patens*, an extant representative of the earliest land plant lineages. *P. patens* was found to contain a large number of ESYTs, distributed over all previously established classes and an additional class not present in *A. thaliana*. Motif discovery identified regions in the Synaptotagmin-like mitochondrial (SMP) domain that may explain phylogenetic relationships as well as protein function. The adaptation mechanisms of *P. patens* necessary to conquer land and its simple tissue structure make it highly suitable as a model organism to study ESYT functions in tip growth, stress responses, and plasmodesmata-mediated transport, and open new directions of research regarding the function of MCSs in cellular processes and plant evolution.

## 1. Introduction

Extended Synaptotagmins (ESYTs) are integral membrane proteins of the endoplasmic reticulum (ER) that play an essential role in establishing and maintaining membrane contact sites (MCSs) [1,2,3]. These specialized regions are sites of close appositions of membranes between the ER and the plasma membrane (PM) or another organelle. ESYTs are conserved across eukaryotes and, due to their tethering function in MCSs, contribute to lipid transfer and calcium homeostasis [4]. In plants, ESYTs have been studied primarily in angiosperms, where they support adaptation to environmental stresses by maintaining the integrity of the PM and regulating signaling pathways [5,6]. Notably, plant ESYT homologs have been implicated in MCSs at plasmodesmata, the intercellular channels that connect neighboring plant cells and are crucial for communication, cell-to-cell transport of metabolites, signals, and even pathogens, and are essential for coordinated tissue development in plants with complex body plans. At plasmodesmata, ESYT homologs participate in immune responses, underscoring the multiple functions of ESYTs in angiosperms.

This review explores the ESYTs of *Physcomitrium patens*, a model bryophyte representing one of the ancient lineages of land plants. The ESYT family of *P. patens* is compared with those of two other bryophytes, *Sphagnum fallax* and *Marchantia polymorpha,* as well as to the angiosperm *Arabidopsis thaliana* and the well-studied ESYT homologs of *Homo sapiens* and *Saccharomyces cerevisiae*. Due its key position in plant evolution, *P. patens* serves as a model organism for studying the mechanisms that enabled plants to transition from aquatic to terrestrial environments such as tip growth, stress responses, and signaling pathways [7,8]. It therefore provides an ideal system for investigating the roles of ESYTs in membrane dynamics at MCSs in the cell and in plasmodesmata-mediated intercellular processes, and sheds light on the functional diversification of ESYTs in land plant evolution. Additionally, *P. patens* is an easily accessible system in research, based on its high rate of homologous recombination, simple life cycle, and well-annotated genome [9,10].

## 2. ESYTs Function as Tethers at Membrane Contact Sites

### 2.1. Membrane Contact Sites

The transport of lipids, proteins, and other cargo is primarily facilitated by vesicle trafficking, which supports cellular growth and maintenance [11,12]. However, cells also employ an alternative mechanism that allows the direct exchange of metabolites and signaling molecules at MCSs between membrane compartments [13]. MCSs were first observed at the interface between the ER and the PM [14], and subsequent studies revealed that the ER interacts with all major organelles, including mitochondria, chloroplasts, lysosomes, and the Golgi apparatus [15,16,17,18,19].

**Figure 1:**
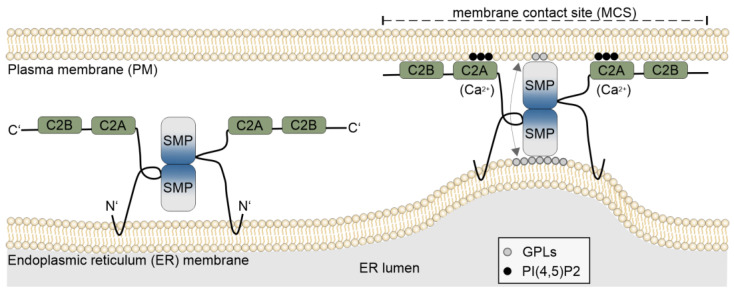
Schematic drawing of Extended Synaptotagmins. ESYT dimers are anchored by their N-terminus to the endoplasmic reticulum (ER), while Ca^2+^-dependent membrane-targeting domains (C2: green box) at the C-terminus can bind to the plasma membrane (PM), depending on the presence of Ca^2+^ ions and the local lipid composition of the PM [54]. Attachment of ESYTs to the PM forms tethers that reduce the distance between the two membrane compartments, creating a membrane contact site (MCS). The two Synaptotagmin-like Mitochondrial-lipid-binding Protein (SMP: gray–blue gradient box) domains form a dimer by interacting in an antiparallel configuration at their respective C-terminal end and associating at their N-terminal regions with the ER and the PM, allowing the transfer of lipids between the ER and the PM, causing changes in the lipid composition at the MCS. One such example is the transfer of glycerophospholipids (GPLs), e.g., phosphatidylethanolamine, phosphatidylcholine, and phosphatidylserine from the ER to the PM, but ESYTs can exchange lipids in both directions.

MCSs enable direct communication, lipid exchange, and regulation of ion dynamics in signaling pathways [13]. They are characterized by the close apposition between two membranes, without resulting in membrane fusion [20]. These sites are tethered by proteins that physically connect the two membrane compartments and are usually dynamic with rapid turnover [21] (Figure 1). ESYTs belong to an expanding group of proteins that act as tethers at MCSs in plants, animals, and yeast (known as Tricalbins or TCBs) [3,5,22,23]. Other MCS tethers in plants include (i) Vesicle-Associated membrane Protein-associated proteins (VAPs), which play central roles in MCSs by linking the ER with the PM, Golgi complex, or chloroplasts [19,24,25]; (ii) Oxysterol-binding-Related Proteins (ORPs), which mediate MCSs between the ER and autophagosomes or endosomes [26,27]; and (iii) Multiple C2 domain Transmembrane domain Proteins (MCTPs), essential in plasmodesmata formation and stabilization [28]. Beyond its role in connecting the PM to the ER at MCSs, the plant-specific tether NET3C forms a multimeric complex by physically interacting with VAP27 and the Kinesin-light-chain-related (KLCR) protein, and functionally interacting with IQ67 domain proteins. Through these interactions, MCSs are linked to both actin and microtubule filaments, contributing to the maintenance of ER and MCS structure and expanding the physical and functional connection between MCSs and the cytoskeleton [29]. Beyond mechanically connecting membranes, MCSs contribute to organelle dynamics, as has been observed in mitochondrial fission in yeast and mammalian cells [30,31] and in the autophagy of mitochondria in plants [32], topics that are not covered in this review.

A well-documented function of MCSs in yeast, animals, and plants is the exchange of lipids between the ER and PM or between the ER and other organelles [13]. For instance, during cold stress, the ESYT homologs AtSYT1 and AtSYT3 in *A. thaliana* regulate the transport of diacylglycerol (DAG) at ER-PM MCSs, ensuring the maintenance of DAG homeostasis at the PM [33]. Compared to vesicle exocytosis, lipid transfer via MCSs has been proposed to offer the advantage of enabling rapid and localized changes in the lipid composition of the PM due to the short distance between the two membrane compartments [34]. In animals, MCSs regulate calcium homeostasis. Human ESYTs (HsESYTs) organize the clustering of Stromal Interaction Molecule-1 (STIM) [35], a calcium sensor embedded in the ER that oligomerizes and relocates to MCSs upon Ca^2+^ depletion. This relocation leads to the activation of PM-localized Calcium Release-Activated Calcium Modulator 1 (CRACM1, ORAI1) [36,37,38], resulting in Ca^2+^ influx known as store-operated Ca^2+^ entry (SOCE) [39,40].

### 2.2. Domain Structure and Functions of ESYTs

ESYTs are found in all eukaryotes and are structurally characterized by three features: (a) an N-terminal hydrophobic region, (b) a single Synaptotagmin-like Mitochondrial-lipid-binding Protein (SMP) domain, and (c) a variable number of C2, Ca^2+^-dependent membrane-targeting domains first identified in protein kinase C [41] (Figure 1). Based on the presence of C2 domains, ESYTs were named after the Synaptotagmins (SYTs), a protein family predominantly expressed in the nervous system of animals and characterized by two C2 domains [42]. In neurons, SYTs regulate the fusion of synaptic vesicles to the PM during calcium-regulated exocytosis of neurotransmitters, playing a critical role in synaptic transmission [43,44,45,46,47]. SYTs are specific to animals and are absent in yeast, plants, and other organisms.

Unlike SYTs, ESYTs are stably anchored to the outer leaflet of the ER membrane by two N-terminal hydrophobic regions that form a hairpin structure. This configuration leaves the N-terminus and the remaining protein sequence exposed in the cytoplasm (Figure 1), distinguishing them from SYTs, which are bound to vesicles. Structural analysis of HsESYTs revealed the presence of an SMP domain, absent in SYTs, that adopts a cylindrical structure with a hydrophobic core [48,49]. This places ESYTs within the Tubular lipid-binding (TULIP) domain family, which includes prokaryotic and eukaryotic proteins involved in lipid sensing and transport [4,50,51]. Although SMP domains are not required for tethering ESYTs to the PM [3,48,52,53], they can transiently associate with membranes at their N-terminal tip and sense membrane curvature, exhibiting a preference for curved regions of bilayers found in the tubules of the cortical ER [54]. Indeed, ESYT homologs that localize at ER–PM MCSs, such as AtSYT1 and HsESYT1, preferentially accumulate and are anchored at the tubular ER in the cell cortex, placing them in the vicinity of the PM [54,55]. The function of the SMP domains of HsESYTs, ScTCBs [2,50,56], and the Arabidopsis homologs (AtSYTs) [57] is to bind and exchange lipids between the ER and the PM [23,54]. While the mechanism of lipid transfer by SMP domains is not fully understood, a model for HsESYTs describes SMP domains of two HsESYTs forming a homo- or heterodimer in an antiparallel configuration through interaction at their respective C-terminal ends. These dimers then associate with their N-terminal tips at the ER and the PM, respectively (Figure 1) [50]. The SMP domains contain a hydrophobic groove that can bind glycerophospholipids at one membrane, followed by a flipping motion along the vertical axis of the dimer to release the lipids at the other membrane compartment [54]. Whether this mechanism applies to other ESYT homologs remains to be investigated. Structural analysis of AtSYT1 supports a similar dimerization mechanism based on SMP domain interaction at their C-terminal termini [57]. Notably, the lipid selectivity of SMP domains is low; instead, a broad spectrum of anionic glycerophospholipids was shown to be transferred by HsESYTs, ScTCBs, and AtSYTs [23,50,54,57,58].

At the C-terminal end, tandem-arranged C2 domains mediate interaction with the PM, bridging the ER and PM with an average intermembrane distance of approximately 10–30 nm [59,60]. This tethering is reversible and does not result in membrane fusion, a key feature distinguishing MCSs from the vesicle fusion processes regulated by SYTs in neuronal cells during exocytosis. Some C2 domains in ESYTs exhibit Ca^2+^-dependent binding to specific acidic phospholipids [61], such as phosphatidylinositol 4,5-bisphophate (PI[4,5]P2) at the PM, while others lack calcium-binding capability [62]. The number of Ca^2+^ ions bound by ESYT C2 domains varies from two to four [62], similar to the variability observed in some of the C2 domains of SYTs, while other C2 domains possess mutations preventing the binding of Ca^2+^ ions [63]. This variability suggests a complex regulatory mechanism for C2 domain interactions with the PM, possibly involving additional roles beyond Ca^2+^-dependent tethering [3,61].

### 2.3. ESYTs in Arabidopsis thaliana

The initial plant ESYT homologs were identified in *A. thaliana* and were designated as SYTs [5,64] before the comprehensive characterization of the ESYTs in animals. In this review, we follow the established nomenclature for Arabidopsis homologs (AtSYTs and AtNTMC2T6s). Newly identified members in bryophytes, however, will be referred to as ESYTs until further structural and functional data are available for their classification. Plant ESYT homologs share a similar domain structure with their animal and yeast counterparts, with three notable differences: (a) the N-terminal hydrophobic region does not consistently form a hairpin; instead, some ESYT homologs possess a single transmembrane region, resulting in the N-terminal end not being exposed to the cytoplasm; (b) they typically contain only 1–3 C2 domains, compared to the 3–5 in animal or yeast homologs; and (c) the role of Ca^2+^ in regulating C2 domain binding to the PM may differ. Targeting of AtSYT1 to the PM requires both of its C2 domains, as demonstrated by truncation analysis, which showed that PM localization occurs only when both C2 domains are present [60]. This is consistent with the mechanism observed in HsESYTs and ScTCBs, where multiple C2 domains mediate PM attachment [65]. This raises the question of how plant ESYTs with a single C2 domain target the PM. In HsSYT1, which contains two C2 domains, the second C2 domain (C2B) is sufficient for Ca^2+^-dependent binding to phospholipids [66,67]. Another mechanism of PM targeting is seen in HsTMEM24, an SMP domain containing protein with a single C2 domain that is anchored to the ER. Although HsTMEM24 exhibits low sequence homology to ESYTs, it localizes at MCSs, regulating signaling functions in neurons and insulin secretion in pancreatic cells based on a mechanism of Ca^2+^-dependent lipid transport from the ER to the PM [68,69,70]. HsTMEM24 associates with the PM through its polybasic C-terminal region, which interacts with anionic lipids. The binding to the PM is supported but is not dependent on the C2 domain. Notably, HsTMEM24 binds to the PM at low cytosolic Ca^2+^, whereas an increase in Ca^2+^ activates protein kinase C, which in turn phosphorylates residues at the C-terminal polybasic region, thereby inhibiting PM association [71]. It remains to be determined whether plant ESYTs with a single C2 domain rely on similar mechanisms.

In *A. thaliana*, seven ESYT homologs (At*SYT1*–At*SYT7*) have been grouped into four classes through phylogenetic analysis [22], with two At*NTMC2T6*s forming an additional class closely related to At*SYT6* [72]. AtSYTs possess a domain structure similar to animal and yeast homologs consisting of (i) an N-terminal hydrophobic region either consisting of a single transmembrane domain or forming a hairpin structure, (ii) an SMP domain, and (iii) 1–3 C2 domains arranged in tandem at the C-terminus (Table S1) [5,22,72,73,74]. Other proteins containing an SMP and C2 domains, but with additional domains or hydrophobic regions located at the C-terminus, are not included in this review. AtSYTs of *A. thaliana* dimerize, attach to the PM through binding of the C2 domains to phosphoinositides or phosphatidylserine [57,75,76], generate MCSs, and transfer lipids between the ER and the PM similarly to animal and yeast counterparts [3,56]. However, some AtSYTs localize at MCSs between the ER and the trans-Golgi-network (TGN), suggesting additional functions of plant ESYTs [72].

AtSYT1 and AtSYT5 are expressed in all tissues, with AtSYT1 displaying the highest expression of all AtSYTs [77], while other homologs show tissue-specific expression as seen in the expression of AtSYT4 in the phloem [78]. The cellular localization of AtSYTs has been examined using approaches such as fluorescent protein tagging under the endogenous promoter in *A. thaliana*, transient heterologous expression in *Nicotiana benthamiana* leaves, and immunolabeling. The intracellular localization of AtSYT1–AtSYT3, AtSYT5, and AtSYT7 (also known as AtCLB1 or AtNTMC2T4) resembles the localization of HsESYTs and ScTCBs, by displaying a punctate appearance at the cortical region of cells and colocalizing with markers of the ER, PM, and MCSs, confirming their roles in MCSs of the ER-PM interface [5,33,73,75,79]. Although an AtSYT2-GFP fusion protein was initially reported to localize at the TGN [80], later studies using immunofluorescence labeling of anti-AtSYT2 in pollen tubes revealed that AtSYT2 localizes both to the TGN and the PM [79]. AtSYT2 localization at the PM was also supported by AtSYT2-DsRED-E5 and was shown to be delivered to the PM from the TGN through conventional secretion [79]. AtSYT4 is primarily expressed in sieve elements precursors and localizes in the cytoplasm concentrating around the nucleus or at the cell cortex depending on the developmental stage of the phloem [78]. AtSYT6 and AtNTMC2T6s have been shown to localize at the ER and colocalize with the TGN marker Vesicle-Associated Membrane Protein 721 (VAMP721) [81], suggesting roles in the forming of ER-TGN membrane contact sites [72,82].

AtSYT1 not only generates and maintains ER–PM MCSs in Arabidopsis leaf cells but also contributes to a second type of MCSs as a complex with VAP27 [83]. Deletion of AtSYTs has revealed roles in stress responses related to mechanical, cold, and ionic stresses, implicating functions in plasma membrane dynamics and repair mechanisms. AtSYT1 provides tolerance to freeze-induced puncture wounds, with extracellular calcium enhancing this effect, indicating a role in calcium-dependent PM resealing [64]. AtSYT1 expression was upregulated in response to mechanical stress, again suggesting a protective role in PM stability [75].

AtSYTs participate in multiple processes by assembling into homo- and heterodimers and forming complexes with other ESYTs [33,57], similar to HsESYTs and ScTCBs [54,84]. For example, seedlings grown under salt stress were more affected in an At*syt1* knockout mutant, due to increased PM permeability [5]. Fluorescent labeling of MCSs has shown that salt stress induces the relocation of AtSYT1 to the cortical ER. This process is dependent on an increase in PI(4,5)P2 at the PM and leads to an expansion of the area occupied by MCSs. Interestingly, while the cortical cytoskeleton was not required to maintain MCSs, it was essential for AtSYT1 accumulation at these sites [76]. Additionally, stress induced by trivalent lanthanum and gadolinium increased phosphatidylinositol-4-phosphate (PI4P) levels at the PM, resulting in the accumulation of a complex composed of AtSYT1, AtSYT5, and AtSYT7 at ER-PM MCSs [73]. These findings further support the idea that AtSYT relocation is driven by increases in PI4P or PI(4,5)P2 at the PM in response to stress. While AtSYT1, AtSYT5, and AtSYT7 do not directly interact with the mechanosensitive ion channel AtMSL10 as in the case of VAP27, they are functionally related to AtMSL10 [85], a key sensor and regulator of osmotic, salt, and mechanical stress [86]. Under cold stress, AtSYT1 and AtSYT3 bind PI4P at the PM, leading to MCS rearrangement and removal of excess DAG. These AtSYTs showed no selectivity for specific DAG species, and the removal of DAG from the PM was identified as a general mechanism for maintaining lipid homeostasis at the PM in response to abiotic stress [33].

AtSYT1 and AtSYT2 have also been reported to influence pollen germination and pollen tip growth; however, gene knockouts resulted in only mild growth defects [79]. Unlike the essential role of Ca^2+^ in regulating the association of some of the C2 domains to PI(4,5)P2 at the PM [58,61] in animal and yeast ESYT homologs, and HsESYTs functioning as calcium sensors and controlling the Ca^2+^ content of the ER through the SOCE mechanism [87], the role of calcium in plant ESYT homologs appears to be different. The C2A domain of AtSYT1 binds Ca^2+^, which enhances its interaction with phosphatidylserine but is not required for binding PI(4,5)P2 or other types of phosphoinositides at the PM [75,88]. The C2B protein sequence of AtSYT1 does not contain a Ca^2+^-binding signature and was proven experimentally to bind phosphoinositides and phosphatidylserine independent of Ca^2+^ [57,75], implying a different contribution of Ca^2+^ to the interaction of AtSYT1 with the PM compared to ESYT homologs of animals and yeast.

Several AtSYTs localize to MCSs of plasmodesmata with positive or negative effects on immune responses, as reviewed in detail by Benitez-Fuente et al. [6]. Plasmodesmata connect plant cells across their cell walls, forming the symplast [89], and are essential for transporting nutrients, nucleic acids, and signaling in plant tissues, all processes that were fundamental for the development of land plants with complex body plans [90]. Pathogens exploit plasmodesmata to spread from cell-to-cell during infection, using a variety of mechanisms, with some viruses using movement proteins to modify the permeability of the plasmodesmata. The structure of a plasmodesma consists of a cytoplasmic sleeve that is surrounded by the PM and traversed by a narrow ER tubule, the desmotubule [91]. The PM is connected to the desmotubule by protein tethers, including AtSYTs [22,92], and the MCSs they form are important in defense responses. AtSYT1 was shown to physically interact with AtSYT5 and AtSYT7 and to colocalize at MCSs. Triple knockout of At*syt1* At*syt5* At*syt7* caused defects in the morphology of ER tubules and increased the diameter of the ER at the entrance to plasmodesmata, indicating that AtSYT1, AtSYT5, and AtSYT7 play a role in constricting the ER, which is required for desmotubule formation [93]. The triple knockout inhibited cell-to-cell transfer of a movement protein related to the *Tobacco mosaic virus* but not free GFP and suppressed infection rates, indicating that AtSYT1, ATSYT5, and AtSYT7 are used by the virus for their active transport through plasmodesmata [22]. In other cases, AtSYTs have beneficial effects on the immune response, as in the case of AtSYT1, AtSYT4, and AtSYT5, which confer resistance to the bacterial pathogen *P. syringae* [94].

### 2.4. P. patens as a Model Organism for Membrane Dynamics

**Figure 2:**
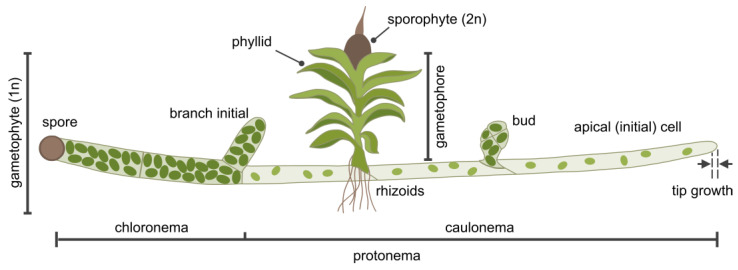
Schematic depiction of *P. patens* development. The haploid gametophyte stage begins with a spore, which, after its first cell division, gives rise to chloronema cells containing numerous large chloroplasts and perpendicular cell walls between neighboring cells. Chloronemata differentiate into caulonemata, which have fewer, smaller chloroplasts and are separated by oblique cell walls. Together, chloronema and caulonema cells form the protonema tissue. The apical (initial) cell of each filament expands through tip growth and is the only cell that divides. Side branch initial cells form new filaments. These cells may develop into filaments identical to the main filament or into buds that grow into gametophores, composed of leaf-like structures (phyllids) and rhizoids. At the center of the gametophore apical region, male and female reproductive tissues develop [7], leading to the formation of the diploid sporophyte upon fertilization. After maturation, the sporophyte releases haploid spores, completing the life cycle of *P. patens*. Not to scale.

*Physcomitrium patens* is a moss bryophyte that is characterized by a dominant haploid gametophyte stage and a short-lived diploid sporophyte stage [7]. Its life cycle begins with haploid spores, which germinate into branched, filamentous protonemata. The apical initial (stem) cells play a dual role in cell proliferation and cell growth. Cell expansion in these apical cells occurs by tip growth [95], a highly polarized form of cell expansion where the growth zone is confined to the apical region of the PM, resulting in elongated cells (Figure 2). Protonemata consist of two cell types that develop sequentially: chloronemata, which primarily function in photosynthesis, and caulonemata, which facilitate the expansion of the colony into suitable habitats and the uptake of nutrients. Chloronemata are the first cells to emerge from spores (or protoplasts). They contain large chloroplasts that are located at the cell periphery and are separated from neighboring cells by cell walls oriented perpendicular to the growth axis [96]. Depending on auxin signaling and environmental cues, chloronemata differentiate into caulonemata [97,98,99]. Compared to chloronemata, caulonemata exhibit faster rates of tip growth and cell division, and neighboring cells are separated by obliquely oriented cell walls [97,99,100]. During the differentiation to caulonemata, substantial cytological changes occur, including the formation of tubular ER, the association of microtubules with the ER, and a reduction in the number and size of chloroplasts [96,101]. Despite having fewer and smaller chloroplasts, caulonemata grow faster than chloronema and may therefore rely on photoassimilates from chloronemata, if no external energy sources such as glucose are available [102,103].

On both chloronemata and caulonemata, side branch initial cells arise, which generate new filaments on chloronemata, while on caulonemata, they either develop new filaments or produce a bud by undergoing a series of programmed longitudinally and transverse cell divisions, depending on hormonal regulation by cytokinin and environmental factors such as light quality [104,105]. Buds develop into gametophores [106], shoot-like structures with leaf-like phyllids, which rely on rhizoids for anchorage and for water and nutrient uptake (Figure 2) [107]. These rhizoids are multicellular filaments arising from epidermal cells at the base or at the mid-stem of the gametophore [108]. Although their cytology and morphology are similar to caulonemata, rhizoids do not branch. Like in protonemata, the apical cell of rhizoids elongates by tip growth and serves as a stem cell, as it is the only cell in each filament that divides [8,108,109]. At the apex of the gametophore, male and female gametangia develop (monoicous plant) that, upon fertilization, produce the diploid sporophyte. The sporophyte undergoes meiosis to produce haploid spores, thereby completing the life cycle.

*P. patens* has been established as a model organism of non-seed plants due to (i) its simple tissue structure, (ii) a well-annotated genome [9], (iii) the predominance of haploid tissues, (iv) a broad range of molecular biology, biochemical, and microscopic methodologies, and (v) a high rate of homologous recombination, comparable to that of the yeast *Saccharomyces cerevisiae*, and genome modifications using highly efficient CRSPR/Cas (clustered regularly interspaced short palindromic repeats) applications [7,110,111]. *P. patens* is especially suited for the study of membrane dynamics due to the evolutionary position of bryophytes between green algae and angiosperms, which possesses ancient features of land plants, and is therefore used to examine the adaptation mechanisms of plant terrestrialization [7].

While plasmodesmata are found in some algae, they exhibit a simple structure without a desmotubule [112]. In bryophytes, plasmodesmata are fully developed and are considered to have been essential in the emergence of land plants [113]. Indeed, the plasmodesmata of *P. patens* share many features found in vascular plants, including angiosperms [113,114], thus providing an ideal system for investigating the role of ESYTs in plasmodesmata. The protonema offers an additional advantage for the investigation of plasmodesmata due to the linear arrangement of chloroplast-rich chloronemata followed by chloroplast-poor caulonemata [115]. Both cell types can be viewed as the two parts of a source–sink model, since caulonemata are believed to rely on the photoassimilates supplied by chloronemata to fulfill their energy requirements [116]. This is further supported by the lack of long-term survival in caulonema-only mutants, whereas viable mutants consisting exclusively of chloronemata have been reported [97,109,117]. Interestingly, while MCSs have not been described yet in *P. patens*, several proteins known to localize at the MCSs of *A. thaliana* have been identified in large-scale proteomics analyses of plasmodesmata [118].

Knowledge of ESYTs in plants is limited to the angiosperm *A. thaliana*. In this review, the putative ESYTs of the bryophytes *Physcomitrium patens*, *Sphagnum fallax*, and *Marchantia polymorpha* will be reported and compared to *Arabidopsis thaliana* in order to provide insight into the ESYT family at an early stage of plant evolution.

### 2.5. ESYTs in Bryophytes

Using the amino acid sequence of either full-length proteins or SMP domains of AtSYTs, AtNTMC2T6, or HsESYT1, the genome databases of *P. patens*, *S. fallax*, and *M. polymorpha* were searched. The identified ESYTs were then used as baits to re-scan the bryophyte genomes in order to find sequences not detected using heterologous ESYTs. A total of 15 *ESYT* genes were identified in *P. patens* (Pp*ESYT1-15*), 15 *ESYT* homologs were found in *S. fallax* (Sf*ESYT1-15*), and 10 *ESYT* homologs were found in *M. polymorpha* (Mp*ESYT1-10*, Table 1). Only sequences containing the typical configuration of ESYTs composed of (i) 1-2 N-terminal hydrophobic region(s), (ii) an SMP domain, (iii) at least one C2 domain, and (iv) no other domains or additional hydrophobic regions at the C-terminus were included for further analysis. Pp*ESYT1-3* [74] and Pp*ESYT1-8* were reported previously [22]. An additional Pp*ESYT* (XP_024382745) was identified previously using the sequence database of the National Center for Biotechnology Information (NCBI) [119]. This *ESYT* differs from Pp*ESYT15*, as identified in this work using the latest annotation of the *P. patens* genome [9], by containing a 17-amino-acid deletion in the SMP domain and a 12-amino-acid insertion in the C2 domain.

**Figure 3:**
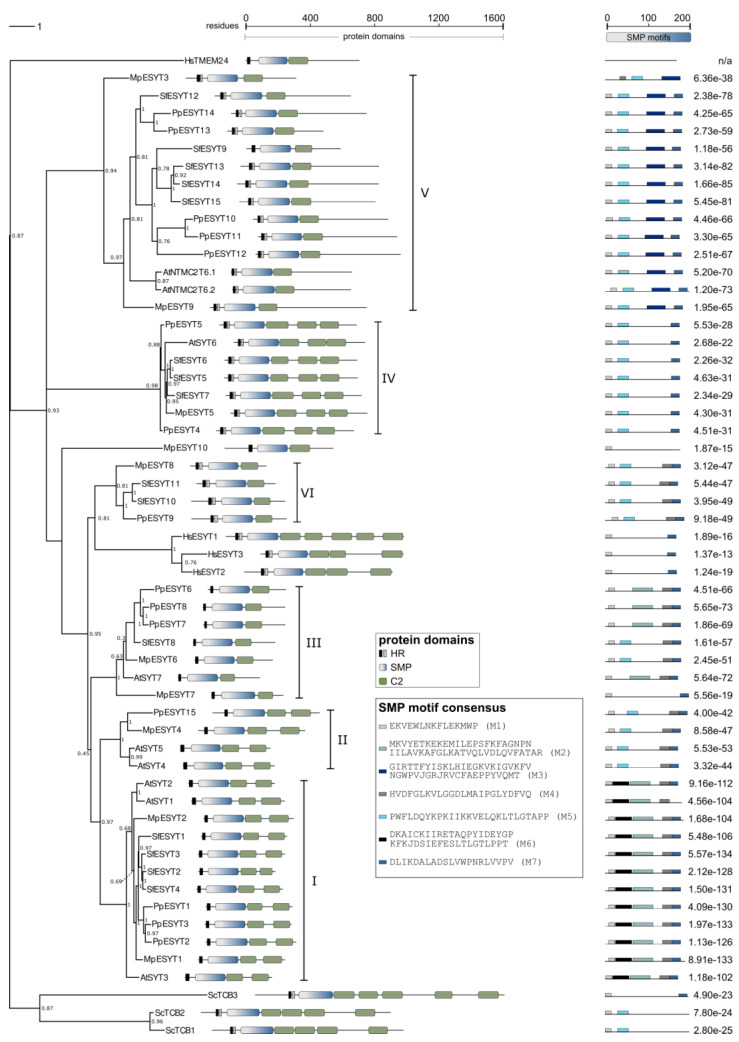
Phylogenetic tree of ESYT homologs. ESYT domain structure and overrepresented SMP motifs are shown. Protein sequences of ESYT homologs and HsTMEM24 of *H. sapiens* [48,68], *S. cerevisiae* [56], and *A. thaliana* [5,22,72] were reported previously. ESYT homologs of *P. patens*, *S. phallax*, and *M. polymorpha* were identified using tBLASTn [125] (protein query against translated nucleotide databases) with genome databases deposited at Phytozome (v13) [126]. The phylogenetic tree was constructed by maximum likelihood and 1000 bootstrap iterations using the phangorn R package [127] (see Supplementary Code S1.R for details). Bootstrap values are shown for the nodes. The branch length bar (upper left scale) represents the average number of substitutions per residue. For a tree containing branch lengths, see Figure S2. The tree was edited using Inkscape [128]. Protein domains were identified using InterPro [129] and SMART [130] analyses. HR: hydrophobic region (black box, single HR; black and gray boxes, hairpin structure), SMP: Synaptotagmin-like Mitochondrial-lipid-binding Protein (gray–blue gradient box), C2: Ca ^2+^-dependent membrane-targeting domain (green box). SMP motifs M1–M7 (shades of gray and blue boxes) were identified using MEME [124], and the corresponding consensus sequences are displayed. Numbers adjacent to motifs depict the combined match *p*-value derived from the product of position *p*-values of the MEME output. Scales indicate amino acid residues of full-length sequences (upper middle scale) or SMP domains (upper right scale). HsTMEM24 could not be aligned in BLASTP or produce motifs in MEME analysis due to low homology to the other sequences; n/a: not applicable.

Multiple sequence alignment (MSA) of the 56 amino acid sequences showed that the sequence lengths vary strongly, with MpESYT7 on one end of the spectrum consisting of 447 residues and ScTCB3 on the other end with 1545 residues (Table S1). The alignment and the consensus sequence show strong divergence between the sequences, even within conserved regions (Figure S1). However, for some positions in the alignment, a strong consensus was detected. At alignment positions 278 and 291, both of which are located in the SMP domain, all analyzed sequences except HsTMEM24 contained tryptophan (W). All sequences shared Asparagine (N) at alignment position 280. These positions, among others, indicate strong conservation and imply functional importance of these residues in the SMPs across all tested phylogenetic groups. The weak sequence homology of HsTMEM24 to all other sequences was expected and is in accordance with its classification outside the HsESYT gene family. Despite this, HsTMEM24 was included in this study because it (i) possesses the domain structure of ESYTs but with a single C2 domain, (ii) forms MCSs between the ER and the PM, and (iii) transfers lipids between the two membrane compartments [69,70]. Some of the putative ESYTs in bryophytes which were identified in this study possessed a single C2 domain and were clustered together with known ESYTs including HsESYTs and ScTCBs, all of which contain at least three C2 domains. This finding, along with the phylogenetic isolation of HsTMEM24 from all other sequences, may indicate that plant-based ESYTs with a single C2 indeed belong to the ESYT family (Figure 3).

The protein sequences of ESYT homologs that we identified in bryophytes were also compared to those of *A. thaliana*, the only ESYT-like family in plants analyzed in detail so far. AtSYTs cluster in four classes [22] while AtNTMC2T6s form a fifth class with a protein structure, cellular localization, and sequence homology related to AtSYT6 [72]. Previous phylogenetic analyses further revealed that class I (AtSYT1, AtSYT2, AtSYT3), class II (AtSYT4, AtSYT5), and class III (AtSYT7) form a clade with a different phylogenetic origin than class IV (AtSYT6) [22]. The ESYT homologs identified in bryophytes were distributed in all five classes and formed an additional class VI that did not contain any AtSYTs (Table 1; Figure 3; see Figure S2 for explicit branch lengths of the phylogenetic tree). In each class, the number of ESYT homologs in bryophytes varied slightly compared to *A. thaliana*, but remarkably, the overall trend seems to be that the ancestors of the first terrestrial plants already possessed ESYT homologs for all classes as identified in *A. thaliana*, with the exception of class VI. Interestingly, class VI contained putative bryophyte ESYT genes for which no homolog exists in *A. thaliana*. These sequences possess two N-terminal hydrophobic regions, probably forming a hairpin structure, an SMP domain, and one C2 domain (Table 1, Figure 3).

The bryophyte *Physcomitrium patens* is a basal land plant and an extant representative of the first plants to succeed in land colonization and form a sister clade to vascular plants. Due to its evolutionary position, *P. patens* is widely utilized to uncover the mechanisms for adaptation of plants on land [120,121]. In our analysis, *P. patens* contained more ESYTs (15) than previously reported and also more than the angiosperm *A. thaliana* [22] (Table 1). The increased number of ESYTs in *P. patens* was consistent with an equal number of ESYT homologs identified in *S. fallax* (15). Interestingly, for *M. polymorpha*, a bryophyte from the clade of liverworts, 10 putative ESYT homologs were identified, comparable to the number of ESYTs in *A. thaliana* (9). In contrast to *P. patens* and *S. fallax*, *M. polymorpha* has not been subjected to whole-genome duplications (WGDs), suggesting that the large ESYT families of *P. patens* and *S. fallax* are partially independent of WGDs. The identification of ESYTs in bryophytes is in agreement with the presence of ESYTs in *Klebsormidium nitens* [22]*,* a terrestrial alga known to exhibit early signs of adaptation mechanisms observed in land plants [122]. The ESYTs of *K. nitens* have been postulated to have participated in the development and function of plasmodesmata, potentially contributing to the adaptation of basal plants to terrestrial conditions [22,122].

**Table 1:** ESYT-like homologs of *Arabidopsis thaliana*, *Physcomitrium patens*, *Sphagnum fallax*, and *Marchantia polymorpha*. Distribution of the number of ESYT and AtNTMC2T6 homologs based on phylogenetic analysis (see Figure 3) in classes I to IV described by Ishikawa et al. [22] and class V reported by Huercano et al. [72]. Class VI did not contain AtSYTs.

	Class I	Class II	Class III	Class IV	Class V	Class VI	Total
*A. thaliana*	3	2	1	1	2	0	9
*M. polymorpha*	2	1	2	1	2	1	9 ^1^
*S. fallax*	4	0	1	3	5	2	15
*P. patens*	3	1	3	2	5	1	15

^1^ MPESYT10 did not belong to classes I–VI.

The structure of the phylogenetic tree, which was constructed using the maximum likelihood method [123], was further supported by the heatmap containing pairwise distances between sequences (Figure S3). It is important to note that the distances were not used for the construction of the tree, but show another aspect of sequence similarity. The figure shows that HsTMEM24 is indeed rather different from all other investigated genes. Also, the genes in class IV show very low sequence similarity to all other putative ESYTs that were analyzed in this study. The genes in class V show lower sequence similarity between each other than the sequences of other classes, like class I, class III, and class IV, indicating a stronger evolutionary divergence than sequences within other classes. This observation is also reflected in the branch lengths of the phylogenetic tree.

The SMP domains of ESYTs play essential roles at MCSs in (i) forming homo- and heterodimers, (ii) sensing the curvature of the membrane bilayer, thus contributing to the localization of ESYTs at the tubular ER of the cell cortex, and (iii) binding and transferring lipids between membrane compartments [4,54,57]. Sequence comparison of the SMP domains of HsESYTs and ScTCBs showed sequence conservation at the C-terminal end of the SMP domains, corresponding to the region of SMP dimerization [50]. In addition to HsESYTs and ScTCBs, the alignment performed in this study included putative ESYT homologs of plants, revealing a few highly conserved residues located at the N-terminal end of the SMP domains, the region of the domain that associates with the PM or the ER (Figure S1). A further comparison of the SMP domains was carried out using the Multiple EM for Motif Elicitation tool (MEME) [124]. The MEME analysis revealed, with a few exceptions, the presence of two conserved motifs M1 and M7 at the N- and C-terminal regions of the SMP domain, respectively (Figure 3). This indicates that the SMP regions needed for interaction with membrane bilayers or dimerization are conserved across the examined phylogenetic groups. The high conservation of the M1 motif at the region of the SMP domain that interacts with different membranes (e.g., the ER, PM, TGN) could imply a low specificity of the SMP binding to the lipid composition of membranes. This is in agreement with the finding that SMP domains are dispensable for the tethering of HsESYTs at MCSs but function as sensors of membrane curvature [54]. The conserved M7 domain located at the interface of SMP dimerization is consistent with the ability of ESYTs to form homo- and heterodimers in animals, yeast, and plants, as well as the participation of the same ESYT in several complexes. This behavior is exemplified by AtSYT1, which, together with AtSYT3, regulates PM integrity in cold stress responses [33], while interacting with AtSYT5 and ATSYT7 in immune responses [22]. Exceptions to the conservation of the M1 and M7 motifs include MpESYT3, which was missing the M1 motif, and AtSYT1, MpESYT10, ScTCB1, and ScTCB2, which lacked the M7 motif at the C-terminus of the SMP domain. HsTMEM24 is the only sequence that does not contain any of the overrepresented motifs in the analyzed SMP domains, providing further evidence for its distinctiveness from the remaining sequences in terms of molecular structure. The core region of the SMP domain, which contains the hydrophobic groove for lipid binding, proves to be more variable than the terminal regions of the domain. The distribution of SMP motif composition throughout the phylogenetic tree aligns with the class formation to some degree. All sequences with SMP motif M6 are found in class I, which contains no other sequences. The same is true for the genes with SMP motif M3, which are part of class V. These observations strongly indicate that the genes within the respective classes, stemming from different species, form orthologs and may therefore share physiological function within the respective organism. Interestingly, almost all classes, with exception of class II and VI, contain genes from all analyzed plant species.

Based on the known functions of the AtSYT homologs present in classes I to IV and the close phylogenetic relationship to the bryophyte ESYTs identified in this study, the physiological importance of the individual identified ESYTs may be hypothesized. The genes in class I could be involved in the tip growth of protonemata and rhizoids, similar to the role of AtSYT1-2 in the tip growth of pollen tubes and root hairs in Arabidopsis. Also, genes belonging to class I to IV may play a role in the restoration of plasma membrane integration posterior to stress exposure as well as in defense mechanisms. The genes belonging to class V, which also contain AtNTMC2T6.1 and AtNTMC2T6.2, have not yet been investigated regarding their biological function. Finally, since for the genes in class VI, no orthologs have been identified in *A. thaliana* according to our analysis, the function of these genes may differ from those observed in *A. thaliana* so far.

## 3. Conclusions

Bioinformatic analysis of the ESYT gene family in the bryophytes *P. patens*, *S. fallax,* and *M. polymorpha* revealed that all ESYT classes of *A. thaliana* were also present in *P. patens*. In addition, an ESYT class unique to bryophytes was identified, with no counterpart in *A. thaliana*, raising the possibility that ESYTs in bryophytes may have similar or also additional roles compared to angiosperms. MCSs and protein tethers at MCSs have not, to our knowledge, been extensively studied in non-seed plants, with the notable exception of the RETICULON1 (RTN1) protein in *M. polymorpha* [131]. MpRTN1 regulates the morphology of ER tubules and interacts with the photoreceptor phototropin at ER–PM contact sites, enabling chloroplast movement in response to high-light or cold conditions [131]. Phototropin also mediates the photoavoidance of chloroplasts in protonemata of *P. patens* [132], which could imply that a similar mechanism of chloroplast movement depending on MCSs may occur in *P. patens*. In *A. thaliana*, ESYTs contribute to PM repair and homeostasis during abiotic stress and regulate plasmodesmata structure and function in defense responses. AtSYT1, in coordination with AtSYT3, regulates plasma membrane homeostasis in *A. thaliana* during salt and osmotic stress and maybe also in drought responses by removing excess diacylglycerol from the plasma membrane [9]. It would be of interest to study ESYTs and their functions in MCSs in *P. patens* and other bryophytes and to determine how they contribute to adaptation mechanisms, such as salt, cold, and water stress, that are present in this early-diverging lineage of non-seed land plants [7,133]. The ability of *P. patens* to cope with drought is one of the striking features of bryophytes that was essential for the ancestral land plants to adapt to conditions outside of the aquatic habitat [134]. Interestingly, each developmental stage of *P. patens* employs distinct mechanisms to adapt to water loss. Both protonemata and gametophores are poikilohydric; however, gametophores additionally possess a protective cuticle against dehydration, whereas sporophytes are homoiohydric, resembling angiosperms [8]. Sporophytes are also the only tissue of *P. patens* bearing stomata, which, unlike those of other plants, accelerate the dehydration of the sporophyte in dry conditions, which precedes the dispersal of the spores [135]. These features, as well as the simple organization of its tissues and extensive protonemal stage, render *P. patens* especially suited for studying ESYTs in tip growth and plasmodesmata function. Future research could illuminate the roles of the genes and conserved motifs identified here in terms of their physiological and biochemical properties in this important model plant and provide information on the role of ESYTs and MCSs in plant evolution.

## 4. Methods

### 4.1. Genome and Protein Database Search

The protein sequences of *H. sapiens* HsESYTs [48], HsTMEM24 [68], and *S. cerevisiae* ScTCBs [2,56] were obtained from the Universal Protein Resource (UniProt, https://www.uniprot.org/ (accessed on 2 December 2024)) [136] and the National Center for Biotechnology Information (NCBI) [119]. ESYT homolog protein sequences AtSYTs and AtNTMC2T6s of *Arabidopsis thaliana* [5,22,33,72,73] were obtained from The Arabidopsis Information Resource (TAIR, https://www.arabidopsis.org/ (accessed on 2 December 2024)) [137]. ESYT homologs were identified in three model bryophytes for which complete annotated genome databases are available at the Phytozome (v13) depository (https://phytozome-next.jgi.doe.gov/ (accessed on 2 December 2024)) [126] using the genome database of *Physcomitrium patens* v6.1 (Phytozome genome ID:870) [9], *Sphagnum fallax* v1.1 (Phytozome genome ID: 522) [138], and *Marchantia polymorpha* v3.1 (Phytozome genome ID: 320) [139]. The databases were searched using as queries the protein sequence of the full-length or the SMP domain of the following sequences: AtSYT1, AtSYT6, AtSYT7, AtNTMC2T6.1, HsESYT1, PpESYT1, PpESYT4, and PpESYT10 (after PpESYT-like sequences were identified in *P. patens*) with the Protein Basic Local Alignment Search Tool (BLASTP) [125,140] applying default settings. Some ESYT homologs of *P. patens* and *M. polymorpha* were reported previously as indicated in Table S1 [22,74].

### 4.2. Protein Domains and Motif Analysis

The protein domains of HsTMEM24 and of ESYT homologs of *H sapiens*, *S. cerevisiae*, and *A. thaliana* were reported previously [2,22,48,56,68,72]. The ESYTs of *P. patens*, *S. fallax*, and *M. polymorpha* were analyzed using DeepTMHMM (v1.0) (https://dtu.biolib.com/DeepTMHMM (accessed on 2 December 2024)) [141] for the prediction of hydrophobic regions. The Integrative Protein Signature Database (InterPro, https://www.ebi.ac.uk/interpro/ (accessed on 2 December 2024)) [129] and Simple Modular Architecture Research Tool (Smart BLAST, https://blast.ncbi.nlm.nih.gov/smartblast/ (accessed on 2 December 2024)) [130] were used for the prediction of protein domains. Only protein sequences with a predicted N-terminal hydrophobic region, an SMP domain, and C2 domain(s) were used for further analyses (Table S1).

The reported and newly identified SMP domains were screened for motifs using MEME analysis (https://meme-suite.org/meme/ (accessed on 2 December 2024)) [124] applying the following settings: motif length from 6 to 50 residues, motif occurrences in all sequences from 2 to 56, a maximum of 1 motif occurrence per sequence, and a maximum of 10 motifs identified. The amino acid sequence of the identified motifs is displayed in Figure 3. The significance of the identified motifs was assessed with the combined match *p*-value, the probability that a random sequence of the same length would yield position *p*-values with a product smaller or equal to that of the tested sequence. A position *p*-value represents the likelihood of a random sequence matching the motif with a score equal or greater to the highest observed in the tested sequence.

### 4.3. Multiple Sequence Alignment and Phylogenetic Tree Construction

The 56 ESYT-like proteins of *H. sapiens*, *S. cerevisiae*, *A. thaliana*, *P. patens*, *S. fallax*, and *M. polymorpha* were subjected to multiple sequence alignment (MSA), using the msa R package (v1.38.0) [142], specifying method = ‘ClustalW’ and the BLOSUM62 [143] substitution matrix. MSA results were visualized with the ggmsa function from the ggmsa R package (v1.12.0) [144,145], and protein domains were added using Inkscape (v0.92.5) [128]. After alignment, a pairwise distance matrix was calculated using the dist.alignment function from the seqinr R package (v4.2-36) [146]. The phylogenetic tree was constructed using the maximum likelihood (ML) approach [123] as implemented in the phangorn R package (v2.12.1) [127]. Model selection was performed using the modelTest function with all 17 available protein models. Inclusion or omission of invariant sites (+I) for completely conserved positions as well as a discrete gamma model (+G(4)) to account for different mutation rates throughout the alignment were tested separately as well as together. Finally, the Jones–Taylor–Thornton [147] model with consideration of invariant sites and varying mutation rates (JTT+G(4)+I) was selected for tree construction as it showed the lowest Bayesian Information Criterion (BIC) of all models with a value of 158,957.4. The ML tree was calculated using the chosen model, and the pml_bb function was used with 1000 bootstrap iterations (rearrangement = “stochastic”). All R code that was used for MSA, phylogenetic analyses, and visualization is provided in the Supplementary Code S1.R script.

## Data Availability

The original contributions presented in this study are included in the article/Supplementary Material. Further inquiries can be directed to the corresponding author.

## Supplementary Materials

The following supporting information can be downloaded at https://www.mdpi.com/article/10.3390/plants14071027/s1, Table S1: List of ESYT homologs used in this study. Figure S1: Multiple sequence alignment of all ESYTs. Figure S2: Phylogenetic tree of ESYTs with branch length distances. Figure S3: Heatmap of pairwise distances between ESYT sequences. CodeS1.R: R code used for multiple sequence alignment, calculation of phylogenetic tree, and visualization of Figure 3 and Figures S1–S3.
