# Supplementary material for: The Extended Synaptotagmins of Physcomitrium patens
Source: Plants (Basel). 2025 Mar 25;14(7):1027. doi: 10.3390/plants14071027 (PMC11990657; doi:10.3390/plants14071027)
Supplement: Supplementary file 1 [file plants-14-01027-s001.zip › FigS1.pdf]

Supplementary Materials

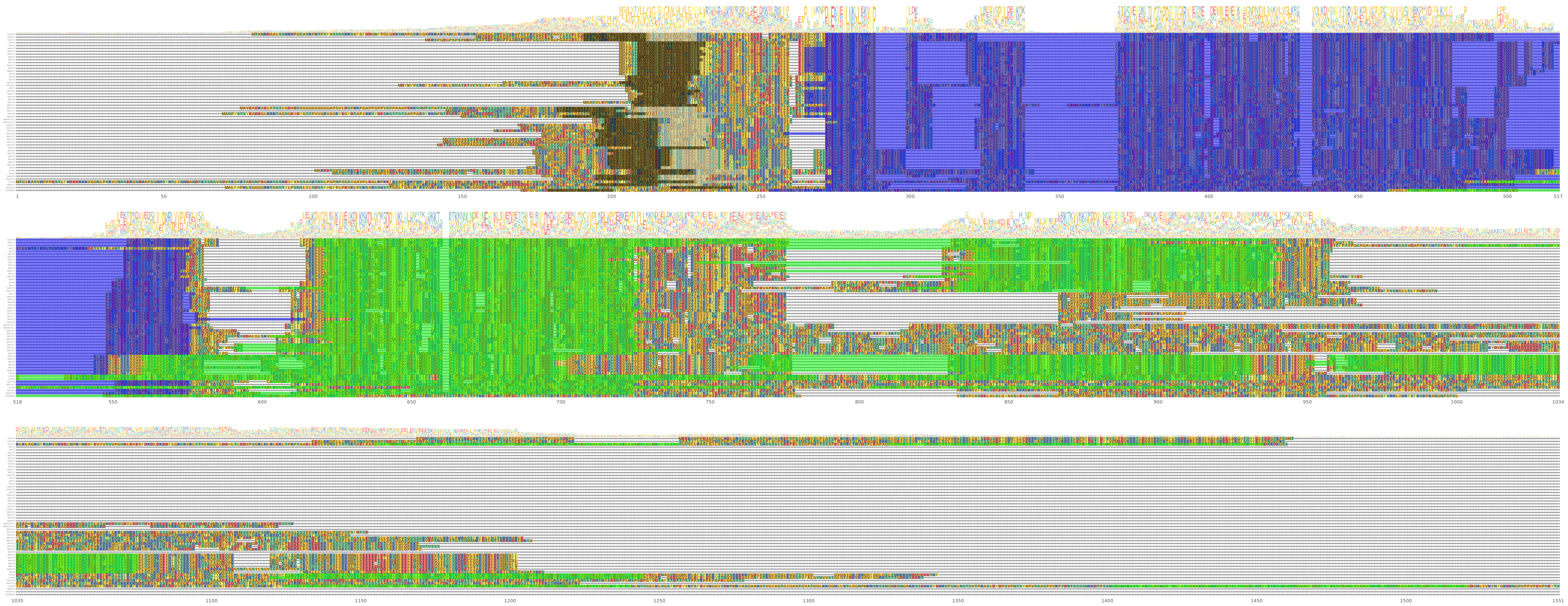

Figure S1. Multiple Sequence Alignment of all ESYT sequences. The x-axis shows the position of the sequences within the alignment. Amino acids are colored according to their side-chain chemistry (blue: positively charged, red: negatively charged; green: polar; yellow: aromatic; orange: hydrophobic and rest). Sequence consensus is indicated by the motif logo (top). Domains identified using the methods mentioned in the main text are shaded (black: HR1; grey: HR2; blue: SMP; green: C2). HR = hydrophobic region; SMP = Synaptotagmin-like mitochondrial-lipid-binding- domain.
