# Supplementary material for: The Extended Synaptotagmins of Physcomitrium patens
Source: Plants (Basel). 2025 Mar 25;14(7):1027. doi: 10.3390/plants14071027 (PMC11990657; doi:10.3390/plants14071027)
Supplement: Supplementary file 1 [file plants-14-01027-s001.zip › FigS2.pdf]

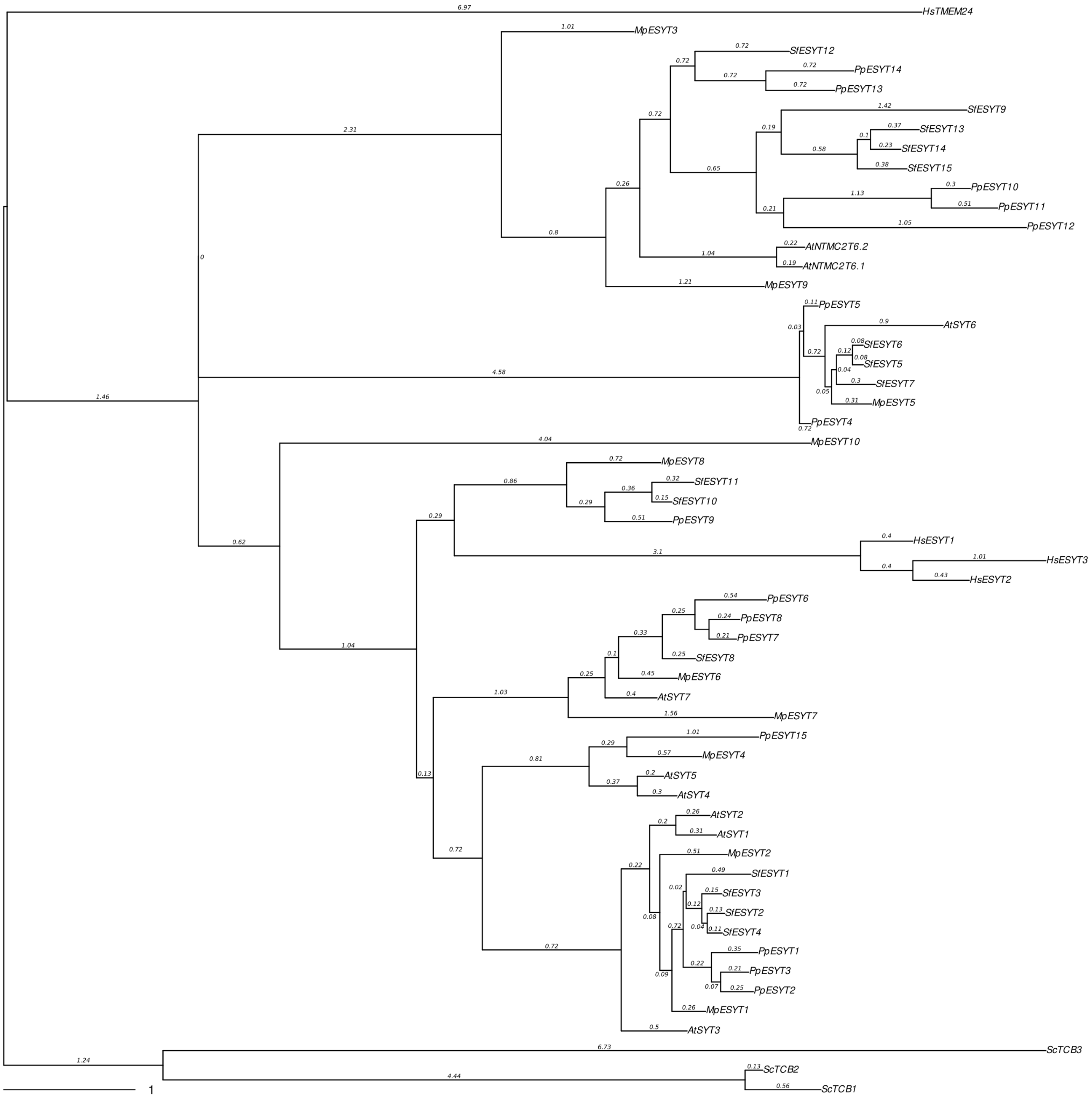


**Figure S2. Phylogenetic tree of ESYTs with branch length distances.** The phylogenetic tree was constructed using Maximum likelihood and 1,000 bootstrap iterations (see Figure 3 for additional information). Bootstrap values are shown for the nodes. Explicit branch lengths (italics) were added. The length of the bar (lower-left corner) corresponds to an average of one amino acid exchange per position.
