# Supplementary material for: The Extended Synaptotagmins of Physcomitrium patens
Source: Plants (Basel). 2025 Mar 25;14(7):1027. doi: 10.3390/plants14071027 (PMC11990657; doi:10.3390/plants14071027)
Supplement: Supplementary file 1 [file plants-14-01027-s001.zip › FigS3.pdf]

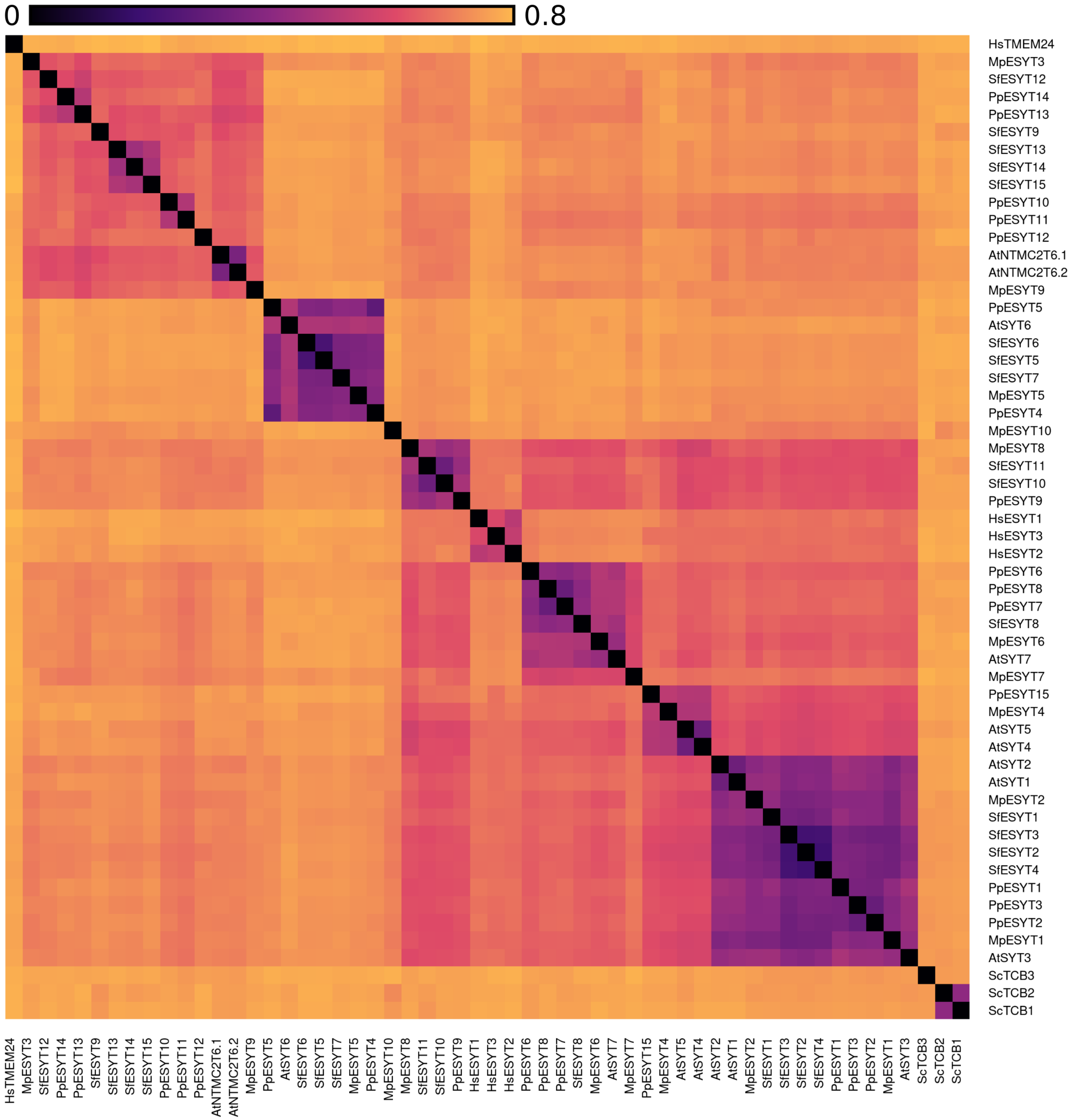

Figure S3. Heatmap of pairwise distances. The pairwise distance between all pairs of ESYTs is shown, with darker colors indicating smaller distances and brighter colors indicating larger distances. The order of labels corresponds to the order in the phylogenetic tree in Figure 3 in the main text and Figure S3.
